# Estimated costs of tuberculosis services in Brazil, 2023

**DOI:** 10.1136/bmjresp-2024-002661

**Published:** 2025-09-14

**Authors:** Elias Jabbour, Marcia Pinto, Ricardo E Steffen, Fernanda Dockhorn, Daniele M Pelissari, Nicole M de Souza, Eduardo de Souza Alves, Anete Trajman, Jonathon R Campbell

**Affiliations:** 1 Department of Medicine, McGill University, Montreal, Québec, Canada; 2 Fernandes Figueira National Institute of Women's, Child, and Adolescent Health, Rio de Janeiro, Brazil; 3 Biomedical Centre, State University of Rio de Janeiro, Rio de Janeiro, Brazil; 4 National Tuberculosis Program, Ministry of Health, Brasilia, Brazil; 5 Federal University of Rio de Janeiro, Rio de Janeiro, Brazil; 6 Department of Global and Public Health, McGill University, Montreal, Québec, Canada; 7 Respiratory Epidemiology and Clinical Research Unit, Centre for Outcomes Research and Evaluation, Research Institute of the McGill University Health Centre, Montreal, Québec, Canada

**Keywords:** Tuberculosis, Respiratory Infection

## Abstract

**Introduction:**

To eliminate tuberculosis (TB) in Brazil, scaling up screening and prevention strategies will be essential. We estimated costs of TB services in Brazil to support budgeting, cost-effectiveness analysis and inform the implementation of these strategies.

**Methods:**

We leveraged databases from five large cities in Brazil (Manaus, Recife, Porto Alegre, São Paulo and Rio de Janeiro) to estimate costs of TB services in 2023 US dollars. We estimated mean costs and 95% uncertainty ranges (95% UR) for specific components and combined these components according to national algorithms for TB diagnosis and treatment in adults and children to estimate costs for different services. We leveraged these outputs to estimate the costs of household contact investigation.

**Results:**

We estimated the mean (95% UR) cost of testing children for TB infection with a tuberculin skin test (TST) or interferon-gamma release assay and providing 3 months of once-weekly isoniazid and rifapentine (3HP) was US$48 ($25–$82) and US$67 ($43–$101), respectively. Providing 6 months of treatment for drug-susceptible tuberculosis (DS-TB) to children was US$557 ($163–$1195). In adults, costs were similar to the cost of TST and 3HP costing US$49 ($25–$86) and 6 months of DS-TB treatment being $583 ($175–$1252). For both children and adults, costs of newer, 6-month treatment regimens for rifampin-resistant tuberculosis (RR-TB) were less expensive than 18-month regimens. In children, the cost was US$4807 ($1559–$10 066) for the 6-month regimen and US$9212 ($2756–$19 567) for the 18-month regimen. Corresponding costs in adults were US$3518 ($1169–$7330) and US$7910 ($2533–$16 717). Across 10 000 households with an index TB patient, we estimated use of a TST and 3HP for TB infection screening and treatment and 6-month regimens for DS-TB and RR-TB disease to cost $1 093 531 (95% UR $409 349–$2 217 054).

**Conclusion:**

There are important cost differences in TB services depending on diagnostic and treatment choices. These data are essential inputs for budgeting and cost-effectiveness.

**WHAT IS ALREADY KNOWN ON THIS TOPIC**

**WHAT THIS STUDY ADDS**

- We estimated the costs of different tuberculosis services in Brazil related to tuberculosis infection and disease screening and treatment.
- Leveraging shorter regimens was estimated to cost less than the use of longer regimens, while the tuberculin skin test was found to be a less costly diagnostic test for tuberculosis infection.

**HOW THIS STUDY MIGHT AFFECT RESEARCH, PRACTICE OR POLICY**

- This study provides costs of a comprehensive package of tuberculosis services collected in a uniform way in order to support budget impact, cost-effectiveness and other evaluations; these will help guide cost-efficient strategies for scaling-up tuberculosis screening and prevention.

## Introduction

 Tuberculosis (TB) is one of the leading causes of death due to a single communicable agent and modelling studies estimate that one-quarter of the world’s population (~2 billion) has been infected with TB.^1^ Approximately 5%–10% of people with TB infection develop TB disease, which requires long treatment regimens (≥6 months). Even after successful TB treatment, survivors are at significant risk of long-term sequelae impacting quality of life and increasing healthcare utilisation.^1^,^3^

Despite having a moderate TB incidence rate in 2023 (49 per 100 000 population), with a population of 211 million, Brazil is among the top 30 high-TB-burden countries.^1^ In Brazil, TB services are offered free of charge by the Brazilian public health system (*Sistema Único de Saúde*).^4^ Nevertheless, TB incidence in Brazil has been increasing since 2016. Innovative strategies to prevent and care for TB are needed.

Evidence suggests TB incidence can be reduced with case finding, treatment and prevention activities.^5^ Their efficient scale-up and deployment, however, require an understanding of their cost. Previous studies have compared costs of specific diagnostic tools and/or treatment regimens,^6^,^8^ but none have considered the complete package of TB services offered in Brazil. Combining costs from several studies for budgeting or cost-effectiveness analyses also poses issues due to methodological heterogeneity in the costing approach and the cost components considered. A contemporary study compiling the costs of a comprehensive list of TB services would therefore serve as a useful resource to policymakers. To support budgeting and cost-effectiveness analyses, and provide key inputs to inform the implementation of TB prevention strategies, we estimated the costs of various TB services in Brazil.^9^

## Methods

### Setting

Brazil is in South America and had a population of 211 million people in 2023. With a gross domestic product per capita of $9281 US dollars (USD), and a Gini index of 0.52 in 2022, Brazil is an upper-middle-income country, yet characterised by significant social and economic inequalities.^10 11^

TB care is provided free of charge in Brazil. On average, 55% of diagnoses and 60% of treatments are carried out in primary care facilities, with the remainder in specialised services and hospitals. Funding of primary care human resources and infrastructure, as well as procedures, is shared across federal, state and municipal levels of government. At the federal level, partial reimbursement for procedures is done according to schedules defined by the *Tabela de Procedimentos, Medicamentos e Órteses, Próteses e Materiais Especiais do Sistema Único de Saúde* (*Tabela SUS*; Table of Procedures, Medications, Orthoses, Prostheses and Special Materials of the Unified Health System) from the Brazilian Ministry of Health.^12^ The federal government also funds the costs of consumables for certain tests, such as interferon-gamma release assays (IGRA) and tuberculin skin test (TST) for TB infection diagnosis as well as Xpert MTB/RIF Ultra cartridges, liquid culture, first-line and second-line drug susceptibility testing (phenotypic) and line probe assays for TB disease diagnosis and drug susceptibility testing. All people requiring drug-resistant TB treatment have their regimens designed and initiated in reference outpatient clinics; treatment follow-up is also done in reference outpatient clinics, though directly observed treatment can be shared with primary care facilities. All drugs are acquired by the federal government and distributed to states, who in turn distribute drugs to the municipalities. Each month, the federal government transfers resources directly to states and municipalities for health surveillance activities, so that these entities can carry out health activities and services in a decentralised manner.^13^ Although the resources above are made available routinely, there is no requirement for states or municipalities to use them specifically for TB services.

Treatment of drug-susceptible TB is done with the global standard of care regimen: 6 months of daily isoniazid and rifampin, supplemented with daily ethambutol and pyrazinamide in the first 2 months with all doses provided in a fixed-dose combination. The tuberculosis preventive treatment (TPT) regimen for TB infection provided to most people is 3 months of once-weekly isoniazid and rifapentine (provided to 60% of those under treatment); 4 months of daily rifampin is provided to a select group of individuals based on age and other risk factors, while 3 months of daily rifampin and isoniazid or mono-isoniazid regimens may also be used. Treatment of drug-resistant TB varies according to drug-resistance patterns.^14^

### Data collection

We prospectively collected costs from the healthcare system perspective using micro costing techniques related to case finding, diagnosis of TB infection and disease, and treatment of TB infection and disease. To determine which costs to collect, we used national and international guidelines and expert opinion from consultants in community clinics across Brazil (box 1). Costs reflect costs of drugs (eg, medications), capital costs (eg, laboratory machines, refrigerators, computers), consumables (eg, gloves, syringes) and personnel costs (salary rates of healthcare providers involved in TB care) (see online supplemental table 1 for detailed information and sources for costs included). We leveraged several databases to obtain cost and health resources information. These included the *Tabela SUS*,^12^ the *Departamento de Informática do Sistema Único de Saúde* (Informatics Department of the Brazilian Unified Health Care System),^15^ the *Banco de Preços* (Price Bank),^16^ the transparency portals of five major cities (Manaus, Recife, Porto Alegre, São Paulo and Rio de Janeiro)^17^ and the Global Drug Facility.^18^ All estimates were reviewed and validated by members of the Brazilian National Tuberculosis Programme (NTP).^19^

Box 1 **Description of the cascade of tuberculosis care and associated actions in Brazil, based on national guidelines^19 21^**

**How are household contacts identified?**

Guidelines are to examine and test contacts of all ages. Individuals should be identified by nurses or doctors when the index patient is diagnosed, by asking the index patient who lives in the same house.

**How are contacts screened?**

All contacts should undergo symptom screening and a tuberculin skin test (TST).*

- Any individual with symptoms of tuberculosis (TB) undergoes a chest X-ray and microbiological testing.
- Asymptomatic individuals <10 years receive a chest X-ray** regardless of TST results.
- Asymptomatic individuals ≥10 years receive a chest X-ray only when TST is positive.

**How is TB infection diagnosed and treated?**

Contacts with a positive TST (or interferon-gamma release assay (IGRA)) and a normal chest X-ray are directly prescribed one of the following TB preventive treatment regimens:

- 3 months of once-weekly isoniazid and rifapentine (treatment of choice for adults ≤50 years).
- 4 months of daily rifampin (treatment of choice for children, adults >50 years and those with liver disease).

Alternative options include:

- 6 months of daily isoniazid.
- 9 months of daily isoniazid.
- 3 months of daily isoniazid and rifampin.

Except for 3 months of once-weekly isoniazid and rifapentine (which has weekly follow-ups), all treatments require monthly follow-ups for pill refill, adherence check and evaluation of side effects. Although not nationally required, some cities implement a regimen under direct observed treatment (DOT) 2–3×/week*.*

**How is TB disease diagnosed?**

To diagnose TB, most small cities use two sputum samples for acid-fast bacilli smear microscopy, whereas large cities (notifying over 70% of TB episodes) collect one sputum sample for Xpert MTB/RIF Ultra testing. Sputum culture is prioritised for retreatment, contacts of drug-resistant TB, those with rifampin-resistance detected on Xpert MTB/RIF Ultra (and subsequently confirmed with phenotypic drug-sensitivity tests), in prisons, Indigenous populations and for people who do not convert smear after 2 months of treatment. When an index patient is identified, a household contact investigation is initiated.

**How is TB disease treated?**

The standard of treatment of rifampin-susceptible TB disease is 6 months of daily rifampin and isoniazid supplemented by daily pyrazinamide and ethambutol in the first 2 months. When resistance is detected, another regimen is specifically prescribed at a tertiary TB centre (see online supplemental table 1). All treatment regimens include monthly medical visits, DOT treatments (3×/week during intensive phase and 2×/week during maintenance phase) and monthly smears.

*While TST is the current standard of screening, IGRA is alternatively implemented for people living with HIV who are contacts or with a CD4/mm³ >350, children 2–10 years, transplant candidates and people about to start immunosuppressive drugs. People living with HIV with a CD4/mm³ below 350 do not require a TB infection test to initiate treatment.**Chest X-rays are typically interpreted by radiologists. Computer-aided detection of TB disease is restricted to research contexts only in Brazil.

### Analysis

To estimate costs associated with the time requirements of different healthcare providers involved in diagnosis and treatment of TB, we calculated mean salaries of personnel using salaries reported in federal, state and municipal transparency portals, as well as reports of average salaries from social organisations in Manaus, Recife, Porto Alegre, São Paulo and Rio de Janeiro in 2023. We calculated mean salaries as the weighted mean of salaries which reflect the proportion of healthcare providers employed by federal, municipal and third-party organisations (ie, *organização social*) (see online supplemental table 2).

For all capital costs, such as the cost of diagnostic equipment, computers and software, we annuitised costs at a standard rate of 3% per year. As federal reimbursement for healthcare services (reported in *Tabela SUS*) is known to be an underestimate of the true costs of delivering these services, we used an inflation factor of 3.51 for all cost estimates derived from *Tabela SUS*, as suggested by Pinto *et al*.^6^

We categorised costs according to specific TB services: (1) household contact investigation and TB infection testing; (2) treatment of TB infection; (3) diagnosis and treatment of drug-susceptible TB disease; and (4) diagnosis and treatment of rifampin-resistant TB disease. We combined costs associated with specific services to arrive at costs of the most recent Brazilian TB algorithms, which included different diagnostics and treatment regimens (box 1). To calculate uncertainty ranges, we estimated gamma distributions for each cost parameter (see online supplemental table 3 for distribution parameters) and used Latin hypercube sampling with 1000 iterations to generate a mean and a 95% uncertainty range (2.5th and 97.5th percentile). We report final costs in 2023 USD using a conversion rate of US$1.00=5.07 Brazilian reals (BRL) as published by the Brazilian central bank for the first two quarters (January–June) of the fiscal year 2023^20^; costs in 2023 BRL are also reported in tables. As diagnosis and treatment options vary between children and adults (weight-adjusted dosages, paediatric formulations of medications and different validated diagnostic tools), we independently estimated costs for some of the services according to different diagnostic and treatment algorithms^19 21^ (box 1).

Finally, to demonstrate potential differences in costs by choices of diagnostic or treatment regimen by age group, we conducted a case study estimating the costs of conducting 10 000 household contact investigations and using different diagnostic and treatment regimens. In this analysis, we used details on the United Nations household composition in Brazil, estimated prevalence of TB infection and TB disease among household contacts, and expected proportion of TB disease that was rifampin-resistant (see online supplemental text 1 for complete details).^22^ Analysis was conducted in R (V.4.1.0).

### Patient and public involvement

We did not involve patients or the public in the design or execution of this study.

## Results

Cost estimates in BRL and USD for TB services are summarised in table 1; see online supplemental table 1 for detailed descriptions. Costs for services organised according to diagnostic and treatment algorithms are reported in table 2.

**Table 1:** Summary of costs of tuberculosis services in Brazil

Components of tuberculosis services	Mean cost USD (95% UR)	Mean cost BRL (95% UR)
**Tuberculosis case finding**		
Symptom screening	2.94 (0.58–7.17)	14.91 (2.94–36.35)
Household contact investigation	2.29 (0.36–5.98)	11.61 (1.83–30.32)
**Tuberculosis infection diagnosis**		
Tuberculin skin test	5.59 (4.56–6.73)	28.34 (23.12–34.12)
Interferon-gamma release assay	24.65 (20.08–29.69)	124.98 (101.81–150.53)
New tuberculosis skin test (Diaskin-Test)	6.11 (4.98–7.35)	30.98 (25.25–37.26)
**Tuberculosis disease diagnosis**		
Xpert MTB/RIF Ultra	20.88 (7.98–48.88)	105.86 (40.46–247.82)
Digital chest X-ray	6.58 (0.65–19.13)	33.36 (3.3–96.99)
Computer aided tuberculosis detection (system, annual)	1058 (27.36–3878.93)	5364.06 (138.72–19 666.18)
Acid-fast bacillus smear microscopy (Ziehl-Neelsen)	2.91 (0.26–8.64)	14.75 (1.32–43.8)
Sputum collection	1.78 (0.01–7.84)	9.02 (0.05–39.75)
Liquid culture	52.68 (34.96–73.84)	267.09 (177.25–374.37)
Solid culture	32.51 (21.6–45.57)	164.83 (109.51–231.04)
First-line drug sensitivity test (four drugs)	183.46 (21.01–518.13)	930.14 (106.52–2626.92)
Second-line drug sensitivity test (three drugs)	173.08 (19.93–485.38)	877.52 (101.05–2460.88)
First-line line probe assay	38.74 (4.44–109.06)	196.41 (22.51–552.93)
Second-line line probe assay	45.28 (5.23–127.49)	229.57 (26.52–646.37)
**Tuberculosis preventive treatment (TPT)** *		
Cost of medical evaluation prior to TPT	4.36 (0.62–11.58)	22.11 (3.14–58.71)
6H treatment (isoniazid daily, 6 months)		
Adult	16.58 (8.33–27.59)	84.06 (42.23–139.88)
Child	19.16 (9.64–31.89)	97.14 (48.87–161.68)
3HR treatment (isoniazid and rifampin daily, 3 months)		
Adult	22.61 (11.40–37.67)	114.63 (57.8–190.99)
Child	14.51 (7.31–24.11)	73.57 (37.06–122.24)
3HP treatment (isoniazid and rifapentine weekly, 3 months)		
Adult	36.74 (10.09–80.52)	186.27 (51.16–408.24)
Child	35.04 (9.60–76.60)	177.65 (48.67–388.36)
4R treatment (rifampin daily, 4 months)		
Adult	46.52 (12.74–101.84)	235.86 (64.59–516.33)
Child	31.43 (8.59–68.89)	159.35 (43.55–349.27)
1HP treatment (isoniazid and rifapentine daily, 28 days)		
Adult	29.86 (3.02–86.91)	151.39 (15.31–440.63)
Child	22.8 (2.30–66.00)	115.6 (11.66–334.62)
6Lfx treatment (levofloxacin daily, 6 months)		
Adult	48.78 (24.57–81.35)	247.31 (124.57–412.44)
Child	28.59 (14.38–47.60)	144.95 (72.91–241.33)
**Tuberculosis disease treatment**		
Standard† drug sensitive tuberculosis treatment		
Adults (6 months)	567.07 (155.63–1242.76)	2875.06 (789.05–6300.80)
Children (6 months)	541.38 (148.20–1184.22)	2744.78 (751.36–6004.02)
Children (4 months, non-severe tuberculosis)	487.43 (134.10–1063.54)	2471.26 (679.88–5392.14)
Rifampin-resistant tuberculosis treatment		
6 months, bedaquiline, pretomanid, linezolid and moxifloxacin (BPaLM)		
Adult	3105.25 (850.62–6796.16)	15 743.62 (4312.64–34 456.53)
Child	4394.48 (1206.38–9618.67)	22 280.01 (6116.35–48 766.66)
Rifampin-resistant tuberculosis treatment		
18 months, standard of care‡		
Adult	7494.76 (2060.08–16 386.49)	37 998.43 (10 444.61–83 079.5)
Child	8797.9 (2397.94–19 258.05)	44 605.35 (12 157.56–97 638.31)

For the purposes of costing, adults are defined as those able to receive the maximum daily dose of a medication, while children are those who will take child-friendly formulations (when available) and are assumed to weigh 25 kg.

* Assumes all complete treatment; costs of incomplete treatment are approximately half.

† Standard drug-sensitive TB treatment includes isoniazid, rifampin, pyrazinamide and ethambutol (daily for 8 weeks, intensive phase) and isoniazid and rifampin (daily for 18 weeks, continuation phase). The 4-month regimen in children shortens the continuation phase of isoniazid and rifampin by 50%.

‡ Based on national recommendations and includes 18 months of levofloxacin, linezolid and terizidone, which is supplemented with bedaquiline in the first 6 months.

§ Recently reported to be efficacious at preventing tuberculosis disease among contacts of rifampin-resistant tuberculosis index patients (Fox *et al*).^31^

BRL, Brazilian reals; 6H, 6 months of daily isoniazid; 1HP, one month (28 days) of daily isoniazid and rifapentine; 3HP, 3 months of once-weekly isoniazid and rifapentine; 3HR, 3 months of daily isoniazid and rifampin; 6Lfx, 6 months of daily levofloxacin; 4R, 4 months of daily rifampin; UR, uncertainty range; USD, US dollars.

**Table 2:** Summary of costs of TB care algorithms in Brazil

Tuberculosis services	Cost (95% UR) in 2023 USD	Cost (95% UR) in 2023 BRL
TB infection testing per household contact		
TST	9.52 (6.59–14.00)	48.29 (33.41–70.97)
IGRA	28.59 (22.83–34.78)	144.93 (115.74–176.34)
TB infection diagnosis and preventive treatment		
Adult		
TST, 3HP	49.17 (24.88–85.70)	249.29 (126.12–434.5)
TST, 4R	57.96 (24.59–114.00)	293.85 (124.66–578)
Child		
TST, 3HP	47.64 (24.66–82.15)	241.52 (125–416.5)
TST, 4R	44.39 (21.36–78.70)	225.04 (108.29–399)
IGRA 3HP	66.70 (42.68–100.66)	338.16 (216.37–510.34)
IGRA, 4R	63.45 (39.28–100.41)	321.67 (199.16–509.08)
TB disease diagnosis		
AFB	15.96 (4.59–35.69)	80.89 (23.25–180.92)
Xpert MTB/RIF Ultra	29.24 (10.41–59.26)	148.23 (52.79–300.43)
Drug-susceptible TB disease diagnosis and treatment		
Adult		
AFB, standard treatment	583 (175–1252)	2956 (889–6347)
Xpert MTB/RIF Ultra, standard treatment	596 (185–1275)	3023 (938–6463)
Child		
AFB smear, short treatment	503 (149–1087)	2552 (753–5512)
AFB smear, long treatment	557 (163–1195)	2826 (825–6061)
Xpert MTB/RIF Ultra, short treatment	517 (163–1094)	2619 (824–5546)
Xpert MTB/RIF Ultra, long treatment	571 (175–1202)	2893 (885–6093)
Rifampin-resistant TB disease diagnosis and treatment		
Adult		
Short (6 months) regimen	3518 (1169–7330)	17 835 (5927–37 161)
Long (18 months) regimen	7910 (2533–16 717)	40 104 (12 842–84 756)
Child		
Short (6 months) regimen	4807 (1559–10 066)	24 371 (7905–51,034)
Long (18 months) regimen	9212 (2756–19 567)	46 704 (13 972–99 207)

For the purposes of costing, adults are defined as those able to receive the maximum daily dose of a medication, while children are those who will take child-friendly formulations (when available) and are assumed to weigh 25 kg.

AFB, acid-fast bacilli; BRL, Brazilian reals; 3HP, 3 months of once-weekly isoniazid and rifapentine; IGRA, interferon-gamma release assay; 4R, 4 months of daily rifampin; TB, tuberculosis; TST, tuberculin skin test; UR, uncertainty range; USD, US dollars.

### Household contact investigation and TB infection testing

Currently, Brazilian guidelines recommend household contact investigations once a TB disease diagnosis is made. Contacts undergo both symptom screening and a TST at a primary care centre, though an IGRA may be used for certain populations (eg, children 2–10 years or people with HIV or other severe immunosuppression). Assuming 2.3 household contacts per index patient according to the United Nations,^23 24^ the cost of identifying and evaluating each contact for TB infection with a TST was estimated to be US$9.52 (95% UR $6.59–$14.00), and with IGRA, it was estimated to be US$28.59 (95% UR $22.83–$34.78). Use of newer antigen-based skin tests had approximately the same cost as TST.

### Treatment of TB infection

According to Brazilian guidelines, any symptomatic contact, any child contact <10 years and all contacts ≥10 years with a positive TST/IGRA are required to receive a chest X-ray to rule out TB disease. If the chest X-ray is normal, they are prescribed TPT. Approximately 80% of those who initiate TPT complete the regimen in Brazil. We estimated that the cost per child 10–17 years with a normal chest X-ray who initiated 3 months of once-weekly isoniazid and rifapentine (3HP) treatment was US$47.64 (95% UR $24.66–$82.15) and who initiated 4 months of daily rifampin (4R) was US$44.39 (95% UR $21.36–$78.70). Alternatively, children <10 years undergo IGRA for screening and accrue higher costs (table 2). Costs for adults (≥18 years) were US$49.17 (95% UR $24.88–$85.70) and $57.96 (95% UR $24.59–$114.00) for 3HP and 4R, respectively.

### Diagnosis and treatment of drug-susceptible TB disease

For individuals with abnormal chest X-rays or other clinical indications of TB disease, sputum is collected for microbiological testing. Based on the laboratory capacity in each city, samples are then sent either for acid-fast bacilli (AFB) smear microscopy (two specimens), or for Xpert MTB/RIF Ultra testing (one specimen). We estimated the cost per person diagnosed using AFB smears to be US$15.96 (95% UR $4.59–$36.59) and with Xpert MTB/RIF Ultra to be US$29.24 (95% UR $10.41–$59.26). For certain populations (see online supplemental table 1), specimens are also prioritised for testing via culture at an additional cost of US$52.68 (95% UR $34.96–$73.84) for liquid culture and US$32.51 (95% UR $21.60–$45.57) for solid culture.

For adults, the standard 6-month regimen for drug-susceptible TB was estimated to have an average cost of US$567.07 (95% UR $155.63–$1242.76). Among children, this cost was US$541.38 (95% UR $148.20–$1184.22). If a shorter 4-month regimen for children with non-severe TB was used, the cost was estimated to be US$487.43 (95% UR $134.10–$1063.54), or a cost reduction of US$54 when compared with the 6-month regimen.

Therefore, for individuals who have an abnormal chest X-ray and ultimately have microbiologically confirmed drug-susceptible TB disease, the overall cost of diagnosing and treating drug-susceptible TB disease with a 6-month regimen using AFB smear as the diagnostic was estimated to be US$583.03 (95% UR $175.25–$1251.90) among adults and US$557.33 (95% UR $162.72–$1195.47) among children, while the cost of using Xpert as the diagnostic was estimated to be US$596.31 (95% UR $185.02–$1274.81) among adults and US$570.61 (95% UR $174.57–$1201.84) among children.

### Diagnosis and treatment of rifampin-resistant TB disease

When rifampin resistance is identified on Xpert MTB/RIF Ultra, or people are failing treatment, an additional sputum sample is collected for first- and second-line drug susceptibility testing. The total cost of these additional tests is US$360.31 (95% UR $101.04–$777.85).

Two regimens are available for treatment of rifampin-resistant TB disease: a short regimen of 6 months or a longer regimen of 18 months. Among adults, the overall cost of conducting additional tests after rifampin-resistance is detected and using the short regimen is US$3517.75 (95% UR $1169.04–$7329.54) while if the long regimen was used, it would be US$7910.06 (95% UR $2533.03–$16 717.08). Similarly, among children, the overall cost with the short regimen is US$4806.89 (95% UR $1559.10–$10 065.86) and with the long regimen is US$9211.76 (95% UR $2755.84–$19 567.48). Taken together, use of the short regimen is associated with a cost reduction of 55.53% (95% UR 53.85%–56.16%) in adults and 47.82% (95% UR 43.43%–48.56%) in children.

### Case study: costs of conducting household contact investigations

* *In our case study, a strategy using TST for TB infection testing, 3HP for TB preventive treatment and shorter regimens for treatment of disease was the least expensive, estimated to have a total cost of $1 093 531 (95% UR $409 349–$2 217 054) when performed across 10 000 household contact investigations (table 3). The most expensive strategy involved IGRA for TB infection testing, 4R for TB preventive treatment and longer regimens for treatment of disease.

**Table 3:** Estimated costs of 10 000 household contact investigation under different strategies

Scenario characteristics			Mean estimated cost per 10 000 households (95% UR)
TB infection test	TB preventive treatment regimen	Length of TB disease treatment*	
TST	3HP	Longer regimens	$1 201 224 ($438 843–$2 452 378)
TST	3HP	Shorter regimens	$1 093 591 ($409 350–$2 217 054)
IGRA	3HP	Longer regimens	$1 639 604 ($795 803–$2 980 458)
IGRA	3HP	Shorter regimens	$1 531 971 ($766 310–$2 745 134)
TST	4R	Longer regimens	$1 483 325 ($457 723–$3 203 814)
TST	4R	Shorter regimens	$1 375 691 ($428 230–$2 968 490)
IGRA	4R	Longer regimens	$1 921 705 ($814 683–$3 731 894)
IGRA	4R	Shorter regimens	$1 814 071 ($785 190–$3 496 570)

Calculations assume guidelines are followed; full description of the scenario is in the Supplement.

* Shorter refers to using the shortest available regimen among those eligible (4 months for drug-susceptible tuberculosis for children, 6 months for drug-susceptible tuberculosis for adults and 6 months for rifampin-resistant tuberculosis in children and adults). Longer refers to using the longest regimen available (6 months for drug-susceptible tuberculosis for children and adults, and 18 months for rifampin-resistant tuberculosis for children and adults).

3HP, 3 months of once-weekly isoniazid and rifapentine; IGRA, interferon-gamma release assay; 4R, 4 months of daily rifampin; TB, tuberculosis; TST, tuberculin skin test; UR, uncertainty range.

## Discussion

We found variations in the costs of TB services based on age—particularly for treatment of rifampin-resistant TB—and based on choice of diagnostics and treatments used. Importantly, we found the costs of identifying and treating TB infection to be substantially lower than the costs of diagnosing and treating TB disease (whether rifampin-susceptible or resistant). This effect is strongly driven by the relatively inexpensive cost of household contact investigation and TPT when compared with the diagnosis and treatment of TB disease.

Compared with TPT, we estimated the costs of treating and diagnosing drug-susceptible TB disease to be 10- to 12-fold higher among adults and 8- to 13-fold higher among children. Despite methodological variations in cost estimates, these findings are similar to those estimated in Canada, where treatment of drug-susceptible TB disease is 15-fold more expensive than diagnosis and treatment of TB infection^25^ as well as in Georgia, where treatment of drug-susceptible TB is 11 times more expensive than diagnosis and treatment of infection. We found a similar benefit of averting rifampin-resistant TB. We estimated that in Brazil, the cost of treating rifampin-resistant TB is 14-fold more expensive than drug-susceptible TB. In the USA, it is 9-fold more expensive,^26^ in Canada 10-fold more expensive^25^ and Georgia 22-fold more expensive.^27^

Our findings further highlight the potential economic impact of prioritising TB prevention. As recently estimated, prompt TB disease diagnosis through systematic screening and provision of TPT could reduce the incidence of TB by ~15% between 2024 and 2050.^28^ Importantly, one-third of this benefit is estimated to come from TPT.^28^ The costs of currently available TB services and costs of different diagnostic and treatment algorithms estimated in this study can be used for budgeting as well as cost-effectiveness analyses, as we have demonstrated in our case study of costing household contact investigations. They can also be leveraged to prospectively estimate costs of expanding both TB disease screening and TPT in Brazil within priority populations, such as people with HIV and household contacts, many of whom are not currently reached by these services.^29^ Given recent global commitments at the United Nations High Level Meeting on TB in 2023 to reach millions with TB disease screening and TPT between 2023 and 2027, our cost estimates are useful to support budgeting these efforts at federal, state and municipal levels.

### Strengths and limitations

Our study has several strengths. We leveraged multiple databases (federal, provincial and municipal) to generate comprehensive cost estimates for every activity surrounding TB care. Information provided by stakeholders within the Brazilian NTP guided the identification of TB care algorithms which supported cost estimates for TB services following Brazilian recommendations. We used data from five major cities in Brazil to systematically estimate costs in a standardised form so that they could be used by decision makers.

Our study is subject to limitations. First, we focused solely on generating cost estimates for different TB services and diagnostic and treatment algorithms. We did not incorporate factors such as diagnostic performance or underlying prevalence of infection or disease when generating these estimates. When testing populations with low pretest probability of infection or disease, these costs may be substantial, but will vary by epidemiological setting. Thus, we provide the key inputs for budgeting and economic evaluation in different epidemiological settings across Brazil and provide a case study for their application. Second, estimates obtained from Federal reimbursement tables are known to be underestimates. While we adjusted these prices with an appropriate factor previously estimated, this may not consistently capture the degree of cost underestimation in federal formularies. Third, salaries of healthcare personnel were obtained from federal and municipal salary transparency tables, as well as third-party organisations, for five major cities whose health expenses are likely higher than smaller cities. Though we used expert opinion to estimate the proportion of healthcare personnel employed through each modality (federal, municipal, third party) in each state to arrive at weighted average salaries, there is heterogeneity in salary and share of personnel employed in each modality across Brazil. Fourth, our study does not capture costs of any additional testing or investigations that might occur when diagnosing and treating TB (such as extensive clinical workups in children), specimen transport costs, nor costs to patients, families or society associated with TB. While the former is difficult to estimate in a standardised manner, these latter costs to patients and families have been estimated in a very recent TB patient cost survey in Brazil.^30^

## Conclusion

In summary, we leveraged several data sources in Brazil to systematically estimate costs of TB services and different diagnostic and treatment algorithms. We found services focused on prevention were the least costly, and those focused on treatment of disease were severalfold more expensive. These essential data are required for economic evaluation and budget impact analysis associated with the scale-up of and prioritisation of TB interventions in Brazil.

## Supplementary material

10.1136/bmjresp-2024-002661 online supplemental file 1

## Data Availability

All data relevant to the study are included in the article or uploaded as supplementary information.
