# Supplementary material for: Estimated costs of tuberculosis services in Brazil, 2023
Source: BMJ Open Respir Res. 2025 Sep 14;12(1):e002661. doi: 10.1136/bmjresp-2024-002661 (PMC12434782; doi:10.1136/bmjresp-2024-002661)
Supplement: online supplemental file 1 [file bmjresp-12-1-s001.docx]

**Supplemental table 1. Cost description of tuberculosis care activities**

| **Item** | **Description** | **Source** |
| --- | --- | --- |
| **Tuberculosis Case Finding and HIV** | | |
| **Symptom Screening** | Cost of a medical visit according to federal reimbursement combined with the cost of a physician or nurse to perform evaluation of symptoms (4.5mins). Time estimate sourced from Alsdurf et al (2020), time cost estimated through real salaries collected in Manaus, Porto Alegre, Sao Paolo, Recife, and Rio in 2023 | [1, 2] |
| **Household Contact Investigation** | Cost of a nurse or physician (average) performing over-the-phone contact investigation (7.5 mins). Time estimates sourced from Alsdurf et al (2020), time cost estimated through real salaries collected in Manaus, Porto Alegre, Sao Paolo, Recife, and Rio in 2023 | [1, 2] |
| **Tuberculosis Infection Diagnosis** | | |
| **Tuberculin Skin Test** | Cost of a single medical visit (assuming TST is done in conjunction with another visit), time taken by nursing staff to administer and read the test (7.4 mins) sourced from Alsdurf et al (2020), reagents (including PPD) and materials required. Costs from Rio de Janeiro 2023 | [1, 2] |
| **Interferon-Gamma Release Assay** | Cost as reported by the National TB Program in Brazil based on purchase records; converted to USD using direct exchange rates. Costs also included consumables, materials, and laboratory technician time. | [3, 4] |
| **Antigen-based Skin Test** | Same non-antigen costs of the TST, except using the dia-skin test reported cost (USD 1.60) in place of tuberculin. Dia-skin test cost has been reported to be consistent over time and no inflation factor applied. | [1, 2] |
| **Tuberculosis Disease Diagnosis** | | |
| **Xpert MTB/RIF Ultra** | Cost of cartridge as reported by National TB Program in Brazil and the Global Fund was of $7.97. Also includes non-cartridge costs as estimated in three representative labs in Pinto et. al. (2015), converted to USD using study reported rate and then inflated using country specific GDP deflator used (2012) - 111% inflation. An additional $0.20 should be added to cost of any activity using Xpert cartridges to account for shipping and insurance costs associated with the procurement. | [3-6] |
| **Chest X-ray** | Federal reimbursement for CXR (2023), with factor adjustment as estimated by Pinto and colleagues (3.51). Converted to USD at a rate of 0.197:1 | [4] |
| **Computer-Aided Detection of Tuberculosis on Chest X-Ray** | InferRead CAD software (one license), with annual costs and annuitized capital costs at 3% over 10 years for software and installation/training, 5 years for laptop, and 3 years for initial maintenance agreement with the next years costing $250 per annum. | [5] |
| **Acid-Fast Bacillus Smear Microscopy** | Federal reimbursement for Ziehl-Neelsen smear (2023), with factor adjustment as estimated by Pinto and colleagues (3.51). | [2, 4] |
| **Liquid Culture** | Cost provided by the National Tuberculosis Program (2023). | [3] |
| **Solid Culture** | Taken from de Almeida et. al. (2017), with cost of $16.50 USD in 2013, inflated using GDP deflator - 97% inflation. | [7] |
| **First-line Drug Sensitivity Testing** | Cost provided by the National Tuberculosis Program (2023). | [3] |
| **Second-line Drug Sensitivity Testing** | Cost provided by the National Tuberculosis Program (2023). | [3] |
| **First-line Probe Assay** | Cost estimated by Figueredo activity-based costing and inflated using GDP deflator from 2022 to 2023 - 8.2%. | [8] |
| **Second-line Probe Assay** | Cost estimated by Figueredo for activity-based costing and inflated using GDP deflator from 2022 to 2023 - 8.2%. | [8] |
| **Tuberculosis Preventative Treatment (TPT)** | | |
| **Medical Evaluation for TPT** | Cost of treatment visit from federal reimbursement list (2023) and a digital chest X-ray. | [2, 4] |
| **6H** | Cost of daily isoniazid (*300mg in adults, weight-adjusted in children*) and monthly medical visits for a total duration of 6 months. | [2-4, 9] |
| **3HR** | Cost of daily isoniazid (*300mg in adults, weight-adjusted in children*) and rifampicin (*600mg in adults, weight-adjusted in children*) and monthly medical visits for a total duration of 3 months. | [2-4, 9] |
| **3HP** | Cost of weekly isoniazid (*900mg in adults, weight-adjusted in children*), weekly rifapentine (*900mg in adults, weight-adjusted in children*), prorated weekly visits for direct observation treatment and monthly medical visits for a total duration of 12 weeks. | [2-4, 9] |
| **4R** | Cost of daily Rifampin (*600 mg in adults, weight-adjusted in children*) and monthly visits for 4 months. | [2-4, 9] |
| **1HP** | Cost of daily isoniazid (*300mg in adults, weight-adjusted in children*), rifapentine (*600mg in adults, weight-adjusted in children*) and 2 medical visits for a total duration of 4 weeks. | [2-4, 9] |
| **6Lfx** | Cost of daily levofloxacin (*1g in adults, weight-adjusted in children*) and monthly medical visits for a total duration of 6 months. | [2-4, 9] |
| **Tuberculosis Disease Treatment** | | |
| **Standard Drug Sensitive Tuberculosis Treatment (RHZE)** | Costs of daily Isoniazid/Rifampin/Pyrazinamide/Ethambutol (*300mg/600mg/1500mg/1200mg in adults or weight-adjusted in children*) plus, 23.3 days hospitalized (prorated for 14% hospitalization rate; 1.5 DOT doses per week (prorated), 1 follow-up per month, 1 smear per month, 3 cultures, and 1 chest x-ray every 4 months. Duration of treatment is 4-6 months. Cost of hospitalization day inflated from 2015 using GDP deflator from 38.40 to 64.90. | [2-4, 9, 10] |
| **Rifampicin Resistant Tuberculosis treatment- Short** | Costs of daily Bedaquiline/Pretomanid/Linezolid/Moxfloxacin (dosages as recommended by WHO). Considers: 26 days hospitalized (on average), 1.5 DOT doses per week (prorated), 1 follow-up per month, 1 smear per month, 3 cultures, and 1 chest x-ray every 4 months. Duration of treatment is 6 months. | [2-4, 9, 10] |
| **Rifampicin Resistant Tuberculosis treatment -Long** | Costs of daily Bedaquiline for 6 months and Pretomanid/Linezolid/Terizidone for 18 months (dosages as recommended by WHO). Considers: 26 days hospitalized (on average), 1.5 DOT doses per week (prorated), 1 follow-up per month, 1 smear per month, 3 cultures, and 1 chest x-ray every 4 months. Duration of treatment is 9-20 months. | [2-4, 9, 10] |

**Supplemental Table 2. Average hourly rate of personnel involved in TB care in Brazil (USD 2023)***

| **Category** | **Overall Average** | **Porto Alegre** | **Manaus** | **Recife** | **Rio de Janeiro** | **Sao Paulo** |
| --- | --- | --- | --- | --- | --- | --- |
| Laboratory Technician | 28 | 51 | 24 | 19 | 21 | 24 |
| Nursing | 68 | 87 | 71 | 62 | 34 | 86 |
| Other healthcare worker | 27 | 36 | 23 | 26 | 19 | 31 |
| Physician | 118 | 73 | 81 | 146 | 127 | 161 |
| Social worker | 54 | 89 | 76 | 33 | 30 | 41 |

**Salary data obtained using a sample of convenience*

**Supplemental Table 3.** Shape and scale parameters for gamma distributions for included costs.

| **Cost Parameter** | **Shape** | **Scale** |
| --- | --- | --- |
| Cost to perform symptom screening per person | 2.861 | 1.028 |
| Cost of household contact investigation per index patient | 2.372 | 0.966 |
| Cost of TST, inclusive of materials and personnel time | 101.074 | 0.055 |
| Cost of IGRA, inclusive of materials | 99.774 | 0.247 |
| Cost of antigen-based skin test, inclusive of materials and personnel time | 101.316 | 0.060 |
| Cost of Xpert per person | 3.259 | 6.407 |
| Cost of HIV test per person | 99.145 | 0.070 |
| Cost of CRP per person | 1.042 | 6.144 |
| Cost of CXR per person | 1.753 | 3.754 |
| Annual cost of CAD license (annuitized); considered in analysis as a new investment and part of implementation in 5% of all healthcare facilities | 15.940 | 66.375 |
| Cost of AFB smear, ZN | 1.681 | 1.731 |
| Cost of sputum collection per person | 0.656 | 2.713 |
| Cost of liquid culture per sample | 27.682 | 1.903 |
| Cost of solid culture per sample | 27.682 | 1.174 |
| Cost of first-line DST per panel | 1.929 | 95.115 |
| Cost of second-line DST per panel | 1.928 | 89.765 |
| Cost of first-line LPA per sample | 1.928 | 20.090 |
| Cost of second-line LPA per sample | 1.929 | 23.467 |
| Cost of pre-TPT evaluation, considering a treatment visit | 2.235 | 1.951 |
| Cost of 6R if complete, adults | 11.129 | 1.490 |
| Cost of 6R if complete, children | 11.115 | 1.724 |
| Cost of 3HR if complete, adults | 11.118 | 2.034 |
| Cost of 3HR if complete, children | 11.111 | 1.306 |
| Cost of 3HP if complete, adults | 4.000 | 9.185 |
| Cost of 3HP if complete, children | 3.998 | 8.765 |
| Cost of 1HP if complete, adults | 1.778 | 16.796 |
| Cost of 1HP if complete, children | 1.777 | 12.832 |
| Cost of 6Lfx if complete, adults | 11.117 | 4.388 |
| Cost of 6Lfx if complete, children | 11.114 | 2.573 |
| Cost of 3HR if incomplete, adults; assumes 50% the price of complete | 7.294 | 1.549 |
| Cost of 3HR if incomplete, children; assumes 50% the price of complete | 11.111 | 0.653 |
| Cost of 6R if incomplete, adults; assumes 50% the price of complete | 11.129 | 0.745 |
| Cost of 6R if incomplete, children; assumes 50% the price of complete | 11.103 | 0.862 |
| Cost of 3HP if incomplete, adults; assumes 50% the price of complete | 4.000 | 4.593 |
| Cost of 3HP if incomplete, children; assumes 50% the price of complete | 4.000 | 4.380 |
| Cost of 1HP if incomplete, adults; assumes 50% the price of complete | 1.776 | 8.406 |
| Cost of 1HP if incomplete, children; assumes 50% the price of complete | 1.779 | 6.409 |
| Cost of 6Lfx if incomplete, adults; assumes 50% the price of complete | 11.117 | 2.195 |
| Cost of 6Lfx if incomplete, children; assumes 50% the price of complete | 11.132 | 1.285 |
| Cost of drug-susceptible TB treatment, adults | 4.000 | 141.748 |
| Cost of 4-month DS-TB treatment, children | 4.000 | 121.745 |
| Cost of 6-month DS-TB treatment, adults | 4.000 | 135.178 |
| Cost of BPaLM RR-TB treatment, adults  (6 months bedaquiline, pretomanid, linezolid, moxifloxacin) | 4.000 | 776.313 |
| Cost of BPaLM RR-TB treatment, children  (6 months bedaquiline, pretomanid, linezolid, moxifloxacin) | 4.000 | 1098.620 |
| Cost of 18-month all oral RR-TB treatment, adults  (6 months bedaquiline, 18 months terizidone, linezolid, levofloxacin) | 4.000 | 1873.690 |
| Cost of 18-month all oral RR-TB treatment, children  (6 months bedaquiline, 18 months terizidone, linezolid, levofloxacin) | 4.000 | 2199.475 |

*Standard deviation estimates informing shape and scale parameters are derived based on proportional standard deviation estimates seen for similar activities in other countries (where available) or by assuming proportional standard deviation (in general, a standard deviation of 25% of the mean).

**Supplemental Text 1. Description of household contact investigation case study**

We used details on the United Nations household composition in Brazil to estimate the average household size and therefore, number of household contacts to be investigated. This estimate was 3.3 household members, resulting in 2.3 household contacts, 17% of whom were <10 years of age [11].

We assumed for each household contact investigation, the cost for the household contact investigation would be incurred, all contacts would have a symptom screen, and all would be tested for TB infection (depending on the scenario, a tuberculin skin test or an interferon-gamma release assay). All children <10y of age would also receive a chest x-ray. Based on a large meta-analysis, we estimated 52% of all contacts would test positive for TB infection and require a chest x-ray, however modeled 60% as this is what was seen in a recent national study in Brazil and within the expectation of the meta-analysis [12]. Of these, we assumed 5% would have an abnormal result and require microbiological testing [13].

We assumed 10% of household contacts had symptoms that could be consistent with TB based on a meta-analysis of cough prevalence and would require a chest x-ray and microbiological testing [14].

Based on current testing patterns, we assumed 70% of household contacts tested for TB disease received Xpert and 30% received two sputum smear tests. We made a simplifying assumption that those with TB disease would be detected by these tests. The overall proportion of people with TB disease among all household contacts was 3.1%, in line with a meta-analysis [12].

Among those positive for TB infection and without disease, we assumed they received treatment according to a specific scenario: 3 months of once weekly isoniazid and rifapentine or 4 months of daily rifampin, assuming 80% completed. Among those treated for disease, we assumed 3.3% had rifampin-resistant disease, based on WHO data, and would receive sputum culture and first and second line phenotypic drug-susceptibility testing [15]. We did not consider other exceptional circumstances where additional testing would be performed. We defined two scenarios and assumed all completed treatment: in the “longer” scenario, adults and children with rifampin-resistant tuberculosis received 18-month regimens and adults and children with drug-susceptible tuberculosis received 6-month regimens. In the “shorter” scenario, adults with drug-susceptible tuberculosis received a 6-month regimen, 67% of children with drug-susceptible tuberculosis would receive a 4-month regimen [16] and 33% would receive a 6-month regimen, and all adults and children with rifampin-resistant tuberculosis would receive a 6-month regimen.

**References**

1. Alsdurf H, Oxlade O, Adjobimey M, Ahmad Khan F, Bastos M, Bedingfield N, et al. Resource implications of the latent tuberculosis cascade of care: a time and motion study in five countries. BMC Health Serv Res. 2020;20(1):341.

2. Brasil. Sistema de Gerenciamento da Tabela de Procedimentos. Ministério da Saúde 2023.

3. Brasil. National Tuberculosis Program. Brasilia: Ministry of Health Brazil; 2023.

4. Pinto M, Entringer AP, Steffen R, Trajman A. Cost analysis of nucleic acid amplification for diagnosing pulmonary tuberculosis, within the context of the Brazilian Unified Health Care System. J Bras Pneumol. 2015;41(6):536-8.

5. Facility GD. Diagnostics, Medical Devices & Other Health Products Catalog. In: Facility GD, editor. Stop TB Parternship. Geneva, Switzerland2023. p. 10-2.

6. Fund TG. New Pricing for Cepheid GeneXpert

Tuberculosis Testing. In: Fund TG, editor. 2023.

7. de Almeida IN, de Assis Figueredo LJ, Soares VM, Vater MC, Alves S, da Silva Carvalho W, et al. Evaluation of the Mean Cost and Activity Based Cost in the Diagnosis of Pulmonary Tuberculosis in the Laboratory Routine of a High-Complexity Hospital in Brazil. Front Microbiol. 2017;8:249.

8. Figueredo LJA, Cesar ALA, Ferrazoli L, Chimara E, Vater MC, Silva S, et al. Cost analysis of GenoType(R) MTBDRplus and GenoType(R) MTBDRsl at the State Laboratory of Sao Paulo, Brazil. Rev Soc Bras Med Trop. 2023;56:e0238-2023.

9. WHO consolidated guidelines on tuberculosis: Module 5: Management of tuberculosis in children and adolescents. WHO Guidelines Approved by the Guidelines Review Committee. Geneva: World Health Organization; 2022.

10. Cortez AO, Melo AC, Neves LO, Resende KA, Camargos P. Tuberculosis in Brazil: one country, multiple realities. J Bras Pneumol. 2021;47(2):e20200119.

11. Nations U. Database on Household Size and Composition 2022 [Available from: <https://www.un.org/development/desa/pd/data/household-size-and-composition>.

12. Fox GJ, Barry SE, Britton WJ, Marks GB. Contact investigation for tuberculosis: a systematic review and meta-analysis. Eur Respir J. 2013;41(1):140-56.

13. Twabi HH, Semphere R, Mukoka M, Chiume L, Nzawa R, Feasey HRA, et al. Pattern of abnormalities amongst chest X-rays of adults undergoing computer-assisted digital chest X-ray screening for tuberculosis in Peri-Urban Blantyre, Malawi: A cross-sectional study. Trop Med Int Health. 2021;26(11):1427-37.

14. Song WJ, Chang YS, Faruqi S, Kim JY, Kang MG, Kim S, et al. The global epidemiology of chronic cough in adults: a systematic review and meta-analysis. Eur Respir J. 2015;45(5):1479-81.

15. WHO. TB Country Profile: Brazil: World Health Organization; [Available from: <https://worldhealthorg.shinyapps.io/tb_profiles/?_inputs_&tab=%22tables%22&lan=%22EN%22&iso2=%22BR%22&entity_type=%22country%22>.

16. Turkova A, Wills GH, Wobudeya E, Chabala C, Palmer M, Kinikar A, et al. Shorter Treatment for Nonsevere Tuberculosis in African and Indian Children. N Engl J Med. 2022;386(10):911-22.
